# Practical Notes and Formulæ

**Published:** 1879-05-20

**Authors:** 


### PRACTICAL NOTES AND FORMULÆ.

**Mild Zinc Ointment.**—Having used in a large number and great variety of diseases the above ointment, we give the formula, which is as follows:

| | | |
|---|---|---|
| ℞ | Ol. olive opt | 2 ounces. |
| | Spermaceti | 1 ounce. |
| | Cera alba | 6 drachms. |
| | Zinc ox. alba | 160 grains. |
| | Acid benzoin | 10 " |
| | Sulph. morph | 10 " |
| | Otto roses | 2 drops. |

The first three articles are to be melted, and while warm the other articles are to be added, after being finely pulverized and mixed together; the whole to stand until its natural consistence is formed.

This ointment can be used in almost every variety of ophthalmic disease, either acute or chronic, in opacity of the cornea, in nebulæ, etc. We have found it very valuable in ophthalmic diseases. It is also very valuable as a local application for tetter and salt rheum; excellent as a dressing for indolent ulcers, as well as wounds in general.—*American Med. Journal.*

**Gentian and Rhubarb Draught.**—

| | | |
|---|---|---|
| ℞ | Infus. rhei | ℥ ss |
| | Tr gentianæ | M. xxx. |
| | Sodii bicarbonatis | gr. x. |
| | Spts. chloroformi | M. x. |
| | Aq. menth. piper qs. ad | ℥ j. M |

**Alkaline Columbo Draught.**—

| | | |
|---|---|---|
| ℞ | Sodii bicarb | gr. x. |
| | Tr. aurantii cort | M. xxx. |
| | Infus. columbæ qs. ad | oz. j. |

The two preceding formulæ are from St. Bartholomew Hospital's pharmacopœia and are said by Dr. T. Lauder Brenton to "work wonders" in cases of chronic gastric catarrh with flatulence—the "windy spasms" of old women.—*New Remedies.*

**Hair Dye without Lead or Silver.**—

No. 1.

| | | |
|---|---|---|
| ℞ | Citrate of bismuth | one ounce. |
| | Rose water | two " |
| | Distilled water | two " |
| | Alcohol | five drachms. |
| | Ammonia, sufficient. | |

No. 2.

| | |
|---|---|
| Hydrosulphate of soda | 12 drachms |
| Distilled water | 4 ounces. |

Each solution is to be applied separately.—*Mich. Med. News.*

**Oxide of Zinc.**—Oxide of zinc is recommended as a specific for the tremor of chronic alcoholism.

**Chloral as a Counter-Irritant.**—Made into a mass with gum tragacanth, spread on paper and applied to the skin, it will produce a blister without pain. Applied as a powder, on cotton, it causes a painful burning sensation. By the former method, a portion is absorbed and the patient falls asleep. Its action is not so uniform as cantharides, but it is a mild vesicant, or an agreeable revulsive. The author quoted would commend such "chloral paper" to physicians, the more so, as it will keep for months without losing its activity if well prepared—*Drug. Circ. and Chem. Gazette.*

**Cough Mixture for Consumption.**—

℞ Morph. sulph.......................... grs. ij.
Acidi sulph. dil.......................... gtt. ij.

Mix et adde

Tr. serpentariæ.......................... ℥ i

Vini antimonii..........................

Vini ipecacuanhæ aa.......................... ʒ ij.

Tr. anisi.......................... ℥ j.

Syr. pruni virgin.......................... ℥ jv.

Mix ft sol.

Dose, a teaspoonful every two or three hours.—*Louisville Medical News.*

**In Chronic Bronchitis.**—

℞ Tinct. benzoin co.......................... ʒ ij.

Pulv. tragacanth.......................... ʒ ss.

Aquæ cinnam.......................... ℥ iij.

Dose, two drams.—*Ib.*

**Dr. Kitchener's Peristaltic Persuaders.**—

℞ Solid ext. of rhubarb.......................... ʒ jss

Oil of caraway.......................... gtt x

Syrup.......................... q. s.

Divide into forty pills. Take two at bed time, and a mild evacuation will follow the next day.

**For Freckles.**—Prof. James C. White, of Howard, recommends the following:

℞ Hydrg. bichloridi.......................... gr vj

Acidi muriatici dil.......................... ʒ i

Aquæ.......................... ℥ iv

Alcohol..........................

Aq. rosæ, aa.......................... ℥ ij

Glycerine.......................... ʒ j

Mix. To be applied carefully at night.—*Michigau Med. News.*

**To Restrain Excessive Secretion and in Hemorrhage.**—

℞ Acidi tannici.......................... gr. v.
Acidi nitrici dil.......................... m vj.
Inf. gentian co.......................... ʒ ij.

—*Louisville Med. News.*

**Toothache Remedy.**—Mr. C. A. Guild writes to the Clinic:

In last week's issue you quote Dr. Lardier on collodion for toothache. I have found collodion mixed with enough carbolic acid to form a jelly-like mass to be an excellent remedy for toothache. About equal parts will form a stiff jelly, which may be taken on one end of a pine stick and placed in the cavity of the aching tooth. The pain will be relieved almost instantly if it depends on an exposed nerve. I have found it the most reliable and convenient remedy I ever tried.

Please dont swallow it.—Ed. *Record.*

**In Syphilitic Skin Diseases, Cachexia, etc.**—

| | | |
|---|---|---|
| ℞ | Pot. iod. | gr. ½-vj. |
| | Sp. am. arom. | m j-v. |
| | Syr. sarsæ. | m x-ʒj. |
| | Aquæ. | ʒij-℥ss or ℥j. |

—*Louisville Med. News.*

**In Irritable Stomach of Young Children, with Vomiting of Sour and Curdled Character.**—

| | | |
|---|---|---|
| ℞ | Pot. Bromid | g. j-iij. |
| | Mist. cretæ. | ʒ j-ij. |
| | Syrupi | q. s. |

—*Ib.*

**A New Hypnotic.**—Prof. H. C. Wood, Jr. has analyzed the seeds of sophora speciosa, a native plant of Texas, and has detected a new alkaloid, which he names *sophoria.* Half of one of the seeds is said to be sufficient to produce delicious exhilaration, followed by a sleep lasting one or two days.—*Hospital Gazette.*

**A Useful Aperient.**—

| | | |
|---|---|---|
| ℞ | Magnesiæ | gr. v–x. |
| | P. rhei | gr. iij–x. |
| | P. cinnam. co | gr. j–iij. |

—*Ib.*

**Laxative Tonic Pills.**—(Dr. Parish.)

| | | | |
|---|---|---|---|
| ℞ | Powdered aloes | gr. xl | |
| | Solid extract rhubarb | ℈ iv | |
| | " " gentian | gr. xl | |
| | Oil of caraway | gtt xij | M. |

Make thirty pills. Dose, one to two pills before dinner.

**To Remove Dry Putty.**—By passing over the putty a hot iron or other metal, it is rendered so soft that it may readily be separated from the wood. This is better than slashing the sash with a sharp knife. —*Drug. Circ. and Chem. Gazette.*

**Capsicum with Quinia.**—Prof. W. V. Thompson says that either capsicum, ginger, or other aromatics, combined with quinia, will diminish the amount required of the latter.—*Md. Med. Journal.*

**Liquor Arsenicalis in Prickly Heat.**—This remedy is highly recommended by a writer in the Indian Medical Gazette. He gives it in the usual doses.

